# Glucose Metabolism Disorders, HIV and Antiretroviral Therapy among Tanzanian Adults

**DOI:** 10.1371/journal.pone.0134410

**Published:** 2015-08-19

**Authors:** Emmanuel Maganga, Luke R. Smart, Samuel Kalluvya, Johannes B. Kataraihya, Ahmed M. Saleh, Lama Obeid, Jennifer A. Downs, Daniel W. Fitzgerald, Robert N. Peck

**Affiliations:** 1 Department of Internal Medicine, Catholic University of Health and Allied Sciences, Mwanza, Tanzania; 2 Department of Internal Medicine, Bugando Medical Centre, Mwanza, Tanzania; 3 Center for Global Health, Weill Cornell Medical College, New York, United States of America; 4 Weill Cornell Medical College in Qatar, Doha, Qatar; FIOCRUZ, BRAZIL

## Abstract

**Introduction:**

Millions of HIV-infected Africans are living longer due to long-term antiretroviral therapy (ART), yet little is known about glucose metabolism disorders in this group. We aimed to compare the prevalence of glucose metabolism disorders among HIV-infected adults on long-term ART to ART-naïve adults and HIV-negative controls, hypothesizing that the odds of glucose metabolism disorders would be 2-fold greater even after adjusting for possible confounders.

**Methods:**

In this cross-sectional study conducted between October 2012 and April 2013, consecutive adults (>18 years) attending an HIV clinic in Tanzania were enrolled in 3 groups: 153 HIV-negative controls, 151 HIV-infected, ART-naïve, and 150 HIV-infected on ART for ≥ 2 years. The primary outcome was the prevalence of glucose metabolism disorders as determined by oral glucose tolerance testing. We compared glucose metabolism disorder prevalence between each HIV group vs. the control group by Fisher’s exact test and used multivariable logistic regression to determine factors associated with glucose metabolism disorders.

**Results:**

HIV-infected adults on ART had a higher prevalence of glucose metabolism disorders (49/150 (32.7%) vs.11/153 (7.2%), p<0.001) and frank diabetes mellitus (27/150 (18.0%) vs. 8/153 (5.2%), p = 0.001) than HIV-negative adults, which remained highly significant even after adjusting for age, gender, adiposity and socioeconomic status (OR = 5.72 (2.78–11.77), p<0.001). Glucose metabolism disorders were significantly associated with higher CD4+ T-cell counts. Awareness of diabetes mellitus was <25%.

**Conclusions:**

HIV-infected adults on long-term ART had 5-fold greater odds of glucose metabolism disorders than HIV-negative controls but were rarely aware of their diagnosis. Intensive glucose metabolism disorder screening and education are needed in HIV clinics in sub-Saharan Africa. Further research should determine how glucose metabolism disorders might be related to immune reconstitution.

## Introduction

In the past decade, antiretroviral therapy (ART) coverage has rapidly expanded to include 7.5 million HIV-infected adults in sub-Saharan Africa (SSA) [1], resulting in a 25% decrease in annual deaths from HIV and longer lifespans for HIV-infected adults [2]. With longer lives and more time on ART, the HIV-infected population in Africa is at increased risk of glucose metabolism disorders (GMDs) [3], and this risk will continue to broaden as African countries implement the 2013 WHO Treatment Guidelines to initiate ART at higher CD4^+^ T-cell counts (CD4 counts) [1].

Published reports have demonstrated a high prevalence of GMDs (20–40%) among HIV-infected adults on ART in SSA [4–6], but only 1 of these studies included HIV-negative and HIV-infected ART-naïve comparison groups [4]. Due to this methodological weakness, it is currently difficult to ascertain if the high prevalence of GMD among HIV-infected adults in SSA is related to HIV infection itself, ART use or simply a high community prevalence of GMDs [7,8]. Additional weaknesses of prior studies from SSA included: 1) most patients were only on ART for short periods of time, 2) single fasting or random blood glucose measurements were often used for diagnosis and 3) inadequate control for between-group differences in age and adiposity.

Therefore, we conducted a cross-sectional study utilizing oral glucose tolerance testing (OGTT) to compare the prevalence and pattern of GMDs in three groups of African adults: an HIV-uninfected group, an HIV-infected, ART-naïve group, and an HIV-infected on ART for ≥2 years group. We hypothesized that the prevalence of GMDs would be at least two times greater among HIV-infected adults on ART for ≥2 years compared with HIV-negative controls and that this difference would not be explained by differences in age, gender or adiposity between groups.

## Methods

### Study design

This was a cross-sectional study designed to compare the prevalence of GMDs among 3 groups: 1) HIV-infected, ART-naïve adults, 2) HIV-infected adults on ART for ≥2 years and 3) HIV-negative controls.

### Study area

The study was conducted in the outpatient HIV clinic of Bugando Medical Centre (BMC) in Mwanza, Tanzania. BMC is the zonal hospital for the Lake Victoria Zone in northwest Tanzania, serving a population of approximately 13 million. The HIV prevalence in the Lake Zone is 6% [9], similar to the national average. The BMC HIV clinic provides care to 3,500 patients of whom 2,700 are currently on ART. Patients are referred to BMC from surrounding community-based voluntary counselling and testing centres in the city of Mwanza. Of note, according to Tanzanian national guidelines, all HIV-infected patients must be assigned a treatment partner who is typically a family member, friend or partner [10]. Also, all adults are routinely screened for diabetes mellitus at time of ART initiation with a fasting blood glucose measurement.

### Study population

All of the study population was recruited from the BMC HIV clinic. The study included 3 groups of adults (all >18 years old) for the purpose of comparisons:

1. HIV-negative adult treatment partners (control group),
2. HIV-infected adults newly establishing care in last 3 months, not yet on ART (HIV-infected, ART-naïve) &
3. HIV-infected adults on ART for ≥2 years (HIV-infected, on ART).

HIV-negative adult treatment partners were chosen as a control group in order to provide a control population with similar socioeconomic status to the 2 groups of HIV-infected adults. All treatment partners who attended the BMC HIV clinic during the study period were eligible for enrolment. Treatment partners were not matched to the HIV-infected adults who were enrolled. Exclusion criteria included pregnancy and failure to attend a follow-up visit on the day after enrolment.

### Study procedure

On the day of enrolment, a revised version of the WHO STEPs questionnaire was administered to determine the prevalence of glucose metabolism disorders and its risk factors [11]. The WHO STEPs questionnaire includes questions regarding diabetes mellitus (testing, diagnosis, and treatment), physical examination, anthropomorphic measurements, and blood pressure according to standard protocols. Additional questions were added regarding HIV diagnosis and treatment.

### Laboratory analysis

At enrolment, blood samples were obtained and the CD4 count was measured using an automated BD FACS Calibur Machine (BD Biosciences, San Jose, CA, USA). A urine pregnancy test was performed on women whose last menstrual period was >1 month prior to the date of interview. Enrolled study subjects were instructed to return to the clinic the following morning after an overnight fast.

All participants underwent an oral glucose tolerance test (OGTT) according to WHO protocol [12]. After an overnight fast for >8 hours, fasting blood glucose levels were measured from a sterile finger prick blood sample using an automated machine (OneTouch Select, LifeScan, CA, USA). The OneTouch Select reports a plasma glucose equivalent and has been shown to be >90% accurate (compared to venous plasma glucose measurement) in diagnosing diabetes mellitus when used for OGTT in resource-limited settings [13]. Each participant was then given 400mls of Lucozade (Glaxo Smith-Kline, London, UK), which is equivalent to a 75g glucose loading dose in water, over a duration of <5 minutes [14]. Blood glucose levels were measured again, 2 hours after ingestion of glucose.

### Definitions

GMD was as defined as the presence of impaired fasting blood glucose, impaired glucose tolerance, or diabetes mellitus, according to the WHO definitions [12]. Impaired fasting glucose (IFG) was defined as fasting blood glucose of 6.1 to 6.9mmol/l (110mg/dl to 125mg/dl) and 2-hour glucose <7.8mmol/l (140mg/dl). Impaired glucose tolerance (IGT) was defined as fasting blood glucose <7.0mmol/l (126mg/dl) and 2-hour glucose ≥7.8 and <11.1mmol/l (140mg/dl and 200mg/dl). Diabetes mellitus (DM) was defined as either a fasting blood glucose ≥7.0mol/l (126mg/dL) or a glucose level ≥11.1mol/l (200mg/dL) 2 hours after a 75g oral glucose load [12].

Hypertension was defined as a sustained elevation of systolic blood pressure (SBP) ≥140 and/or diastolic blood pressure (DBP) ≥90 on 2 different days or current antihypertensive therapy according to the Joint National Committee 7 (JNC-7) definition, and graded according to the same guidelines [15]. Central obesity was defined as a waist/hip ratio of ≥0.85 for women and waist/hip ratio ≥0.90 for men according to the WHO [11].

### Statistical analysis

The primary outcome of the study was the prevalence of GMDs (as defined above). The primary study analysis was to compare the prevalence of GMDs between each HIV-infected group and the HIV-negative control group. According to a recent population-based survey, 1.9% (95% CI, 0.7–5.0%) of adults in Mwanza city have diabetes mellitus while 16.4% (95% CI, 11.7–22.4%) have hypertension (Kavishe BB, Mwanza Interventional Trials Unit, personal communication). We hypothesized that 10% of HIV-infected adults on ART would have glucose metabolism disorders. Using Fisher’s exact test, we calculated that 150 patients in each group would provide 90% power to detect this difference for p<0.05.

Data analysis was done using STATA version 11 (San Antonio, Texas). Descriptive statistics were computed by determining medians (interquartile ranges) for continuous variables and proportions (percentages) for categorical variables. Differences between medians were determined using the rank sum test and differences between proportions were determined using Fisher’s exact test. For ordered categorical variables, the nonparametric test for trend was used. P-values of less than 0.05 were considered significant.

Factors associated with GMDs were determined by logistic regression. Multiple logistic regression models were performed in order to determine whether the relationship between HIV status and GMD could be explained by confounding. All baseline characteristics were evaluated by a pre-determined, minimally-adjusted logistic regression model adjusting for age and sex, since these were expected to differ between groups. Furthermore, additional, pre-determined multivariable analyses were performed to adjust for BMI and waist/hip ratio (since these were the factors most highly expected to explain differences in glucose metabolism disorder prevalence between groups) as well as a fully-adjusted model including all variables with a p-value of <0.05 by minimally adjusted multivariable analysis. BMI and waist-to-hip ratio were not included together in any models due to collinearity. Variables associated with HIV infection and ART use (such as CD4 counts and individual ART drugs) were not included in the multivariable models due to collinearity with the group variables and the smaller number of subjects with these additional variables. For associated factors, odds ratios (OR) were determined with 95% confidence intervals (95% CI). The likelihood ratio test was used to compare the logistic regression models.

### Ethics Statement

The study was approved by the Institutional Review Boards at Bugando Medical Centre and Weill Cornell Medical College. All study participants were informed about the study by a nurse or doctor fluent in Kiswahili and provided written informed consent before participation. All results were made available to clinicians and recorded in the patient’s file. Disease management was conducted by the health care workers of the clinic according to Bugando Medical Centre and Tanzanian management protocols and practices.

## Results

### Enrolment

During the study period, 488 adults were screened and 34 were excluded from the study: 7 patients were found to be pregnant (3 HIV-infected ART-naïve and 4 HIV-infected on ART), and 27 did not return the following day (11 HIV-negative controls, 9 HIV-infected ART-naïve, 7 HIV-infected on ART). A total of 454 adults were enrolled: 153 HIV-negative controls, 151 HIV-infected ART-naïve adults and 150 HIV-infected adults on ART.

### Baseline characteristics

Table 1 describes the baseline characteristics of the 3 groups.

**Table 1:** Baseline characteristics of 454 study participants.

Variable	HIV-negative Control (n = 153)	HIV-infected ART-naïve (n = 151)	HIV-infected on ART (n = 150)
	Proportion (%) Median (IQR)	Proportion (%) Median (IQR)	Proportion (%) Median (IQR)
**Female**	94 (61.4%)	89 (58.9%)	115 (76.7%)
**Age (years)**	38 (32–46)	37 (32–44)	40 (38–47)
**Education level**			
Incomplete primary	27 (17.7%)	25 (16.6%)	30 (20.0%)
Complete primary	82 (53.6%)	98 (64.9%)	94 (62.7%)
Secondary and above	44 (28.8%)	28 (18.5%)	26 (17.3%)
**Work type**			
Manual	109 (71.2%)	114 (75.5%)	105 (70.0%)
Office	44 (28.8%)	37 (24.5%)	45 (30.0%)
**Vigorous work**	15 (9.8%)	21 (13.9%)	33 (22.0%)
**Mode of transportation**			
Walking or bicycle	130 (85.0%)	110 (72.9%)	117 (78.0%)
Motorized vehicle	23 (15.0%)	41 (27.1%)	33 (22.0%)
**Ease of living index** *			
Low	86 (56.2%)	94 (62.3%)	81 (54.0%)
Middle	20 (13.1%)	24 (15.9%)	26 (17.3%)
High	47 (30.7%)	33 (21.9%)	43 (28.7%)
**Ever smoker**	6 (3.9%)	16 (10.6%)	10 (6.7%)
**Current smoker**	5 (3.3%)	4 (2.7%)	0
**Fruit/vegetable servings/week**	6 (4–12)	8 (5–12)	9 (6–13)
**Sugary drinks/day**	1 (0.5–2)	1 (0.5–2)	1 (1–2)
**Current alcohol**			
None	119 (77.8%)	120 (79.5%)	136 (90.7%)
<once/week	19 (12.4%)	16 (10.6%)	8 (5.3%)
≥once/week	15 (9.8%)	15 (9.9%)	6 (4.0%)
**Body mass index (kg/m** ^**2**^ **)**	23.8 (22.3–25.8)	22.0 (20.2–24.3)	23.7 (21.5–27.9)
<18.5	5 (3.3%)	18 (11.9%)	4 (2.7%)
18.5–25	104 (68.0%)	113 (74.8%)	80 (53.3%)
25–30	33 (21.6%)	9 (6.0%)	45 (30.0%)
>30	11 (7.2%)	11 (7.3%)	21 (14.0%)
**Waist/hip ratio**	0.84 (0.82–0.87)	0.84 (0.80–0.89)	0.87 (0.82–0.91)
**Central obesity** **	44 (28.8%)	56 (37.1%)	78 (52.0%)
**CD4 T-cell count (cells/μL)**	NA	215 (150–321)	378 (263–521)
**ART duration (months)**	NA	NA	56 (31–68)
**Protease inhibitor use**	NA	NA	18 (12.0%)

* Ease of living index was defined according to the presence of water, electricity and/or flushing toilets inside the home. Low = 0/3. Medium = 1-2/3. Higher = 3/3

** Defined as waist/hip ratio of ≥0.85 for women and waist/hip ratio ≥0.90 for men

The characteristics of the 3 groups were broadly similar. Notable differences included that HIV-infected adults on ART were slightly older (median age 40 (38–47) vs. 38 (32–46) and 37 (32–44) years in the other 2 groups), more likely to be female (76.7% vs. 61.4% and 58.9%) and had a higher prevalence of central obesity (52.0% vs. 28.8% and 37.1%). HIV-infected, ART-naïve adults had lower mean BMI (22.0 (20.2–24.3) kg/m^2^ vs. 23.8 (22.3–25.8) and 23.7 (21.5–27.9)) and were severely immunosuppressed (mean CD4 T-cell count 215 (150–321) cells/μL vs. 378 (263–521) in the group on ART). HIV-infected adults on ART had been using ART for a median of 56 (31–68) months; 18 (12.0%) were taking protease inhibitors, which was a combination of lopinavir and ritonavir for all 18 of them.

### Glucose metabolism disorders

Table 2 shows the prevalence of glucose metabolism disorders and glucose levels in the 3 groups.

**Table 2:** Glucose metabolism disorders (GMD) among 454 study participants.

Variable	HIV-negative Control (n = 153)	HIV-infected ART-naïve (n = 151)	p-value vs. control	HIV-infected on ART (n = 150)	p-value vs. control
	Proportion (%) Median (IQR)	Proportion (%) Median (IQR)		Proportion (%) Median (IQR)	
**Any GMD** *	11 (7.2%)	12 (8.0%)	0.83	49 (32.7%)	**<0.001**
**Diabetes mellitus**	8 (5.2%)	1 (0.7%)	**0.04**	27 (18.0%)	**0.001**
**Impaired fasting glucose**	0	5 (3.3%)	**0.03**	1 (0.7%)	0.50
**Impaired glucose tolerance**	3 (2.0%)	6 (4.0%)	0.33	21 (14.0%)	**<0.001**
**Fasting blood glucose (mmol/L)**	4.7 (4–5)	4.7 (4–5.1)	0.55	5.6 (4.8–6)	**<0.0001**
**2-hour blood glucose (mmol/L)**	5.6 (5–6)	5.5 (5–6.1)	0.41	6.6 (5.8–8.1)	**<0.0001**

* Primary outcome: defined as either Diabetes Mellitus or Impaired Fasting Glucose or Impaired Glucose Tolerance

Overall, GMDs were more common among HIV-infected adults on ART (32.7% vs 7.2% of HIV-uninfected controls, p<0.001). DM (18.0% vs 5.2% among controls, p = 0.001) and impaired glucose tolerance (14.0% vs. 2.0% of controls, p<0.001) were also more common among HIV-infected adults on ART. The prevalence of GMDs among HIV-infected, ART-naïve adults was similar to that of controls (8.0% vs. 7.2%, p = 0.83). Only one HIV-infected, ART-naïve adult had DM (0.7% vs. 5.2% of controls, p = 0.04).

Of the 30 total study subjects with IGT in our study, 11/30 had fasting blood glucose >6 mmol/L and therefore would have been labelled as IFG by the WHO definition used in this study if only fasting blood glucose measurements were obtained and OGTT had not been performed. Of these 30 study subjects with IGT, 26/30 had fasting blood glucose >5.6 mmol/L and therefore would have been labelled as IFG by the definition of the American Diabetes Association [16]. The median fasting blood glucose for those patients with IGT was 6.0 (5.9–6.3) mmol/L vs. 4.8 (4–5.2) mmol/L versus for those without IGT.

### Glucose testing, awareness and treatment

The number of subjects who reported prior glucose testing was <25% in all 3 groups: 33/153 (21.6%) in the control group, 16/151 (10.6%) in the HIV-infected, ART-naïve group and 18/150 (12.0%) in the HIV-infected, on ART group. Rates of awareness, treatment and control were low. Of 36 subjects with DM, only 2 (including 1/1 in the HIV-infected, ART-naïve group and 1/27 (3.7%) in the HIV-infected, on ART group) were aware of their diagnosis. None of the subjects with DM were controlled according to the IDF definition [17].

### Factors associated with GMD

In the partially-adjusted logistic regression model of all factors listed in Table 3, only age (OR = 1.03 (1.003–1.06) per year), ease of living index (OR = 2.83 (1.46–5.47) for middle vs. low), BMI (OR = 1.07 (1.01–1.13) per unit BMI), waist-hip ratio (OR = 1.07 (1.02–1.12) per 0.01 ratio units), central obesity (OR = 2.04 (1.20–3.49)), and current CD4 (OR = 1.001 (1.000–1.003) per unit CD4) were associated with GMDs. Of note, neither ART duration (OR = 1.01 (0.997–1.030) per month on ART), protease inhibitor use (OR = 1.44 (0.51–4.03)), nevirapine use (OR = 1.18 (0.56–2.48)), efavirenz use (OR = 0.81 (0.40–1.63)), tenofovir use (OR = 0.94 (0.46–1.95)), stavudine use (OR = 1.25 (0.62–2.50)), nor zidovudine use (OR = 0.60 (0.30–1.20)) were associated with GMDs.

**Table 3:** Factors associated with glucose metabolism disorders (GMD) among 454 Tanzanian adults by logistic regression adjusted for age and sex.

Variable	No GMD (n = 382)	GMD (n = 72)	Odds Ratio (95%CI)
	Proportion (%) Median (IQR)	Proportion (%) Median (IQR)	
**Female**	249 (65.2%)	49 (68.1%)	1.29 (0.74–2.25)
**Age (years)**	38 (33–46)	40 (36–48.5)	**1.03 (1.003–1.06)**
**Education level**			
Incomplete primary	71 (18.6%)	11 (15.3%)	1
Complete primary	226 (59.2%)	48 (66.7%)	1.48 (0.72–3.04)
Secondary and above	85 (22.3%)	13 (18.1%)	1.16 (0.48–2.82)
**Work type**			
Manual	275 (72.0%)	53 (73.6%)	1
Office	107 (28.0%)	19 (26.4%)	0.93 (0.52–1.66)
**Vigorous work**	55 (14.4%)	14 (19.4%)	1.54 (0.79–3.00)
**Mode of transportation**			
Walking or bicycle	301 (78.8%)	56 (77.8%)	1
Motorized vehicle	81 (21.2%)	16 (22.2%)	1.04 (0.56–1.92)
**Ease of living index**			
Low	228 (59.7%)	33 (45.8%)	1
Middle	51 (13.4%)	19 (26.4%)	**2.83 (1.46–5.47)**
High	103 (27.0%)	20 (27.8%)	1.40 (0.76–2.56)
**Ever smoker**	26 (6.8%)	6 (8.3%)	1.32 (0.50–3.50)
**Current smoker**	9 (2.4%)	0	
**Fruit & vegetable (servings/week)**	8 (5–12)	9.5 (6–12)	1.03 (0.99–1.07)
**Sugary drinks/day**	1 (0.5–2)	1 (1–2)	0.99 (0.82–1.19)
**Current alcohol**			
None	312 (81.7%)	63 (87.5%)	1
<once/week	36 (9.4%)	7 (9.7%)	0.91 (0.38–2.16)
≥once/week	34 (8.9%)	2 (2.8%)	0.30 (0.07–1.30)
**Body mass index (kg/m** ^**2**^ **)**	23.1 (21.1–25.3)	23.7 (21.1–28.3)	**1.07 (1.01–1.13)**
<18.5	24 (6.3%)	3 (4.2%)	1
18.5–25	256 (67.0%)	41 (56.9%)	1.16 (0.33–4.07)
25–30	69 (18.1%)	18 (25.0%)	1.98 (0.53–7.37)
>30	33 (8.6%)	10 (13.9%)	2.01 (0.49–8.23)
**Waist/hip ratio per 0.01 increase**	0.84 (0.81–0.89)	0.87 (0.83–0.92)	**1.07 (1.02–1.12)**
**Central obesity** *	139 (36.4%)	39 (54.2%)	**2.04 (1.20–3.49)**
**CD4 T-cell count (cells/μL**)** **per 1 cell/μL increase**	285 (182.5–407)	350 (246–518)	**1.001 (1.000–1.003)**
<200	69 (28.8%)	8 (13.1%)	1
200–350	86 (35.8%)	22 (36.1%)	1.97 (0.82–4.75)
350–500	47 (19.6%)	14 (23.0%)	2.09 (0.79–5.52)
>500	38 (15.8%)	17 (27.9%)	**3.11 (1.19–8.16)**

* Defined as waist/hip ratio of ≥0.85 for women and waist/hip ratio ≥0.90 for men

** For all 301 study subjects with HIV including 61 with GMD and 240 without GMD

We also performed multiple, pre-determined multivariable logistic regressions to determine if the higher rates of GMDs among HIV-infected, on ART could explained by other confounders known to be associated both with HIV status and GMDs. The results of these models are shown in Table 4. In the unadjusted model, being HIV-infected and on ART (for ≥2 years) was associated with a 6-fold increased odds of having a GMD (OR = 6.26 (3.10–12.63), p<0.001). Correcting for other possible confounders such as age, sex, BMI and ease of living index did not change this estimate. Adjusting for waist-hip ratio slightly decreased the OR of association to ≈5.7 in 2 models, but the association remained highly statistically significant (p<0.001 for all models listed in Table 4).

**Table 4:** Multivariable models for association between HIV status and glucose metabolism disorders (GMD) to assess for confounding.

Model	HIV-negative control (n = 153)	HIV-infected ART naïve (N = 151)	p-value vs. control	HIV-infected on ART (N = 150)	p-value vs. control	Likelihood ratio test (compared to unadjusted model)
**Unadjusted**	1	1.11 (0.48–2.61)	0.80	**6.26 (3.10–12.63)**	**<0.001**	**0**
**Adjusted for age + sex**	1	1.16 (0.49–2.73)	0.73	**6.30 (3.09–12.86)**	**<0.001**	**1.97, 2 d.f.**
**Adjusted for age + sex + body mass index (BMI)**	1	1.23 (0.52–2.89)	0.64	**6.16 (3.02–12.58)**	**<0.001**	**3.90, 3 d.f.**
**Adjusted for age + sex + waist/hip ratio (WHR)**	1	1.09 (0.46–2.58)	0.84	**5.68 (2.76–11.67)**	**<0.001**	**5.59, 3 d.f.**
**Adjusted for age + sex + BMI + Ease of Living Index**	1	1.25 (0.53–2.96)	0.61	**6.20 (3.04–12.70)**	**<0.001**	**4.88, 4 d.f.**
**Adjusted for age + sex + WHR + Ease of Living Index**	1	1.12 (0.47–2.65)	1	**5.72 (2.78–11.77)**	**<0.001**	**6.42, 4 d.f.** *

All models are compared to HIV-negative controls. Models 1, 2, 3 & 4 were predetermined based on most likely confounders. Models 5 + 6 included other baseline characteristics significantly associated with glucose metabolism disorders in the minimally-adjusted model.

* Best fit mode

### Association between GMD and hypertension

There was a strong and dose-dependent association between GMDs and grade of hypertension among HIV-negative adults but this association was not statistically significant among HIV-infected adults. See Table 5 for details.

**Table 5:** Association between glucose metabolism disorders (GMD) and grade of hypertension in the 3 study groups.

	Normal	Prehypertension	Hypertension	P-value for trend
**GMD among 153 HIV-negative adults (controls)**	1/66 (1.5%)	2/62 (3.2%)	8/25 (32.0%)	**<0.001**
**GMD among 151 HIV-infected ART-naïve adults**	4/69 (5.8%)	6/74 (8.1%)	2/8 (25.0%)	0.17
**GMD among 150 HIV-infected adults on ART**	13/49 (26.5%)	18/58 (31.0%)	18/43 (41.9%)	0.14

## Discussion

In our study in Tanzania, 1/3 of HIV-infected adults on ART for ≥2 years were found to have GMDs, a prevalence that was 4x higher than among HIV-negative study subjects and similar to HIV-infected adults in the US and Europe [18,19], and nearly 20% had frank DM. Although a systematic review in 2011 found no association between HIV or ART and GMDs in SSA [20], this review included data mainly from cross-sectional and case-control studies and conceded that most adults in these studies had been on ART for only short periods of time (most <2 years). Two later published cohort studies from SSA with study populations similar to our own were not included in that systematic review, and found a higher prevalence of GMD among HIV-infected patients after initiation of ART [6,21]. Zannou investigated FPG trends in a cohort of 88 HIV-infected patients followed from ART initiation until 24 months into therapy. During the first 24 months of therapy the prevalence of GMDs increased from a baseline prevalence of 3.8% to a high of 39.2% at follow up (IFG 3.8% to 31.6%, DM 0 to 7.6%). We identified a similar prevalence in our cross-sectional study. Similar results have been seen among HIV-infected adults on ART in the US [19,22]. Prior cross-sectional studies may have underestimated to the true prevalence of GMDs among adults on long-term ART because only 2 other studies of GMDs among HIV-infected adults in SSA used OGTT and both of these excluded patients with known GMDs [23,24].

In our study, the prevalence of all GMDs among HIV-infected, ART-naïve adults was low, similar to the prevalence among HIV-negative controls. Data are few regarding the prevalence of GMDs among HIV-infected, ART-naïve adults in SSA. One study from Italy demonstrated IFG was more common among HIV-infected, ART-naïve adults compared to HIV-negative controls [18], and the Multicenter AIDS Cohort Study in the USA showed that the prevalence and incidence of diabetes was higher among HIV-infected adults compared to controls [19,22], but the baseline CD4^+^ T-cell count was higher in both these groups of HIV-infected adults compared to our study. Study results from SSA have been mixed. Mercier and Ngatchou found that there was a higher prevalence of GMDs among adults who were HIV-infected, ART-naïve compared to HIV-negative controls [25,26], but 3 additional studies from SSA showed no difference in average glucose measurements or GMDs between HIV-infected, ART-naïve adults and HIV-negative controls [4,27,28]. Of note, all of these studies used fasting glucose alone; since OGTT was not performed in these others studies, IGT could not be diagnosed leading to an underestimation of the total burden of GMD.

Disturbingly, very few HIV-infected adults with diabetes mellitus were aware of their diagnosis. In fact, among HIV-infected adults on ART with diabetes mellitus, only one (<5%) knew that they had diabetes mellitus. Similarly low rates of DM awareness have been reported in other studies from Africa [29,30], but one might hope that awareness rates would be higher in the context of regular healthcare visits for HIV care. It is possible that, since our current standard of care is to screen HIV-infected adults for DM at the time of ART initiation, clinicians had been lulled into a false sense of security by the low prevalence of diabetes mellitus detected at that time. Clearly GMD screening and treatment must be integrated into HIV care in clinics in SSA, but such integration will be challenging given the limited resources of low-level health facilities in our region for diabetes diagnosis and management [31]. On the other hand, successful integration of HIV and DM management in lower level health facilities in SSA could improve the acceptability and quality of care for both of these diseases, as previously demonstrated with other conditions [32].

The higher prevalence of GMDs among HIV-infected adults on ART was not explained by the most likely confounders such as difference in waist-hip ratio, BMI, age, gender and socioeconomic status. Even after adjusting for differences in age, gender, adiposity and socioeconomic status, HIV-infected adults on ART were still found to have a nearly 6-fold greater odds of having GMD compared to the HIV-negative control patients. The most likely alternative explanation is that GMDs may be a direct result of ART use since some, but not all [18], studies from high income countries have identified an increased prevalence of GMDs with cumulative exposure to ARTs [19,33,34]. In our study GMDs were not significantly associated with duration of ART in general or with PI use specifically. Due to the relatively small sample size of our study and the small number of different drug combinations (including PI-based regimens), our study may have lacked power to detect such associations. Still, the fact that the higher prevalence of GMDs among HIV-infected adults on ART in our study could be explained neither by obvious confounders nor by ART duration leaves the reason for the higher prevalence of GMDs in this group open to conjecture.

The higher prevalence of GMDs among HIV-infected adults on ART may be related to dysregulated inflammation in the setting of severe immunosuppression and subsequent immune reconstitution. In our study a higher current CD4 was associated with GMDs in both univariable and minimally adjusted analyses; HIV-infected adults with a current CD4 >500 had a 3-fold greater odds of GMDs compared to those with a CD4 <200, and a clear dose-response was seen. Our findings seem to fit with those recently reported from a prospective randomized ART-initiation trial in the United States and Puerto Rico, in which the development of higher fasting plasma glucose levels over a 96-week period on ART was significantly associated with higher baseline HIV-1 RNA levels [35]. Other data from the same study demonstrated that higher baseline C-reactive protein (CRP) was significantly associated with subsequent development of non-AIDS-defining clinical events (diabetes, cardiovascular disease, cancer, pneumonia) [36].

Our study has limitations. Since this study is cross-sectional, we cannot make any conclusions as to causality. Nevertheless, this study does generate some interesting hypotheses as to the higher prevalence of GMDs among HIV-infected adults on ART. In addition, our HIV-negative control group may not be representative of the general population, although the prevalence of diabetes mellitus was similar to a recent representative population survey in our city. Further, haemoglobin A1C and HIV viral load testing are not currently available at our hospital. Also, additional data that may have been useful, such as family history of diabetes, hepatitis C status, and weight change from baseline, were not available for our study subjects.

## Conclusions

In conclusion, HIV-infected Tanzanian adults on ART had a 4-fold higher prevalence of GMDs than HIV-negative control patients. One-third of these adults had some GMD, 20% had diabetes mellitus and 15% had IGT. Among the 27 with diabetes mellitus who had completed at least two years of ART, only one was aware of their diagnosis and the remaining 26 were asymptomatic. In contrast, the prevalence of GMDs among HIV-infected, ART-naïve adults was low, similar to HIV-uninfected adults, and the only subject with diabetes mellitus was already diagnosed. The higher prevalence of GMDs among HIV-infected adults on ART could not be explained by differences in adiposity, age, gender or socioeconomic status but were associated with CD4^+^ T-cell counts. Clearly, diabetes mellitus screening and management must be integrated into routine HIV care. In addition, further studies are needed to determine the pathophysiologic explanation for the higher prevalence of GMDs among HIV-infected adults on ARTs and whether this may be related to dysregulated inflammation in the setting of immune reconstitution.

## References

[1] World Health Organization (2013) Global update on HIV treatment 2013: results, impact and opportunities. Geneva, Switzerland: World Health Organization Available: http://www.who.int/hiv/pub/progressreports/update2013/en/

[2] Joint United Nations Programme on HIV/AIDS (UNAIDS) (2013) Global report: UNAIDS report on the global AIDS epidemic 2013. Geneva, Switzerland: UNAIDS. Available: http://www.unaids.org/en/media/unaids/contentassets/documents/epidemiology/2013/gr2013/unaids_global_report_2013_en.pdf

[3] Narayan KMV, Miotti PG, Anand NP, Kline LM, Harmston C, Gulakowski R, et al HIV and Noncommunicable Disease Comorbidities in the Era of Antiretroviral Therapy: A Vital Agenda for Research in Low- and Middle-Income Country Settings. J Acquir Immune Defic Syndr. 2014;67 Suppl 1: S2–7. 10.1097/QAI.0000000000000267 25117958

[4] Awotedu K, Ekpebegh C, Longo-Mbenza B, Iputo J. Prevalence of metabolic syndrome assessed by IDF and NCEP ATP 111 criteria and determinants of insulin resistance among HIV patients in the Eastern Cape Province of South Africa. Diabetes Metab Syndr Clin Res Rev. 2010;4: 210–214. 10.1016/j.dsx.2010.09.002

[5] Berhane T, Yami A, Alemseged F, Yemane T, Hamza L, Kassim M, et al Prevalence of lipodystrophy and metabolic syndrome among HIV positive individuals on Highly Active Anti-Retroviral treatment in Jimma, South West Ethiopia. Pan Afr Med J. 2012;13: 43 23330034 PMC3542806

[6] Zannou DM, Denoeud L, Lacombe K, Amoussou-Guenou D, Bashi J, Akakpo J, et al Incidence of lipodystrophy and metabolic disorders in patients starting non-nucleoside reverse transcriptase inhibitors in Benin. Antivir Ther. 2009;14: 371–80. 19474471 10.1177/135965350901400307

[7] Petersen M, Yiannoutsos CT, Justice A, Egger M. Observational Research on NCDs in HIV-Positive Populations: Conceptual and Methodological Considerations. J Acquir Immune Defic Syndr. 2014;67 Suppl 1: S8–S16. 10.1097/QAI.0000000000000253 25117964 PMC4317266

[8] Ali MK, Magee MJ, Dave JA, Ofotokun I, Tungsiripat M, Jones TK, et al HIV and Metabolic, Body, and Bone Disorders: What We Know From Low- and Middle-Income Countries. J Acquir Immune Defic Syndr. 2014;67 Suppl 1: S27–39. 10.1097/QAI.0000000000000256 25117959

[9] Tanzania Commission for AIDS (2013) Tanzania HIV/AIDS and Malaria Indicator Survey 2011–12. Dar es Salaam, Tanzania: Tanzanian Ministry of Health.

[10] National AIDS Control Programme (NACP) (2009) National Guidelines for the Management of HIV/AIDS, 3rd edition Dar es Salaam, Tanzania: Tanzanian Ministry of Health.

[11] World Health Organization (2011) WHO STEPS Instrument. Geneva, Switzerland: World Health Organization.

[12] World Health Organization (2011) WHO STEPS Instrument. Geneva, Switzerland: World Health Organization Available: hhttp://apps.who.int/iris/bitstream/10665/43588/1/9241594934_eng.pdf

[13] Priya M, Mohan Anjana R, Pradeepa R, Jayashri R, Deepa M, Bhansali A, et al Comparison of capillary whole blood versus venous plasma glucose estimations in screening for diabetes mellitus in epidemiological studies in developing countries. Diabetes Technol Ther. 2011;13: 586–91. 10.1089/dia.2010.0218 21406012

[14] Goff LM, Frost GS, Hamilton G, Thomas EL, Dhillo WS, Dornhorst A, et al Carbohydrate-induced manipulation of insulin sensitivity independently of intramyocellular lipids. Br J Nutr. 2003;89: 365–75. 10.1079/BJN2002789 12628032

[15] Chobanian AA V, Bakris GL, Black HR, Cushman WC, Green L a, Izzo JL, et al Seventh report of the joint national committee on prevention, detection, evaluation, and treatment of high blood pressure. Hypertension. 2003;42: 1–104. 14656957 10.1161/01.HYP.0000107251.49515.c2

[16] American Diabetes Association. Diagnosis and classification of diabetes mellitus. Diabetes Care. 2014;37 Suppl 1: S81–90. 10.2337/dc14-S081 24357215

[17] International Diabetes Federation. Global Guideline for Type 2 Diabetes: recommendations for standard, comprehensive, and minimal care. Diabet Med. 2006;23: 579–93. 10.1111/j.1464-5491.2006.01918.x 16759299

[18] Bonfanti P, Giannattasio C, Ricci E, Facchetti R, Rosella E, Franzetti M, et al HIV and metabolic syndrome: a comparison with the general population. J Acquir Immune Defic Syndr. 2007;45: 426–31. 10.1097/QAI.0b013e318074ef83 17514013

[19] Brown TT, Cole SR, Li X, Kingsley LA, Palella FJ, Riddler SA, et al Antiretroviral therapy and the prevalence and incidence of diabetes mellitus in the multicenter AIDS cohort study. Arch Intern Med. 2005;165: 1179–84. 10.1001/archinte.165.10.1179 15911733

[20] Dillon DG, Gurdasani D, Riha J, Ekoru K, Asiki G, Mayanja BN, et al Association of HIV and ART with cardiometabolic traits in sub-Saharan Africa: a systematic review and meta-analysis. Int J Epidemiol. 2013;42: 1754–71. 10.1093/ije/dyt198 24415610 PMC3887568

[21] Kiage JN, Heimburger DC, Nyirenda CK, Wellons MF, Bagchi S, Chi BH, et al Cardiometabolic risk factors among HIV patients on antiretroviral therapy. Lipids Health Dis. 2013;12: 1–9. 23575345 10.1186/1476-511X-12-50 PMC3641018

[22] Brown TT, Li X, Cole SR, Kingsley LA, Palella FJ, Riddler SA, et al Cumulative exposure to nucleoside analogue reverse transcriptase inhibitors is associated with insulin resistance markers in the Multicenter AIDS Cohort Study. AIDS. 2005;19: 1375–83. 16103768 10.1097/01.aids.0000181011.62385.91

[23] Dave JA, Lambert EV, Badri M, West S, Maartens G, Levitt NS. Effect of nonnucleoside reverse transcriptase inhibitor-based antiretroviral therapy on dysglycemia and insulin sensitivity in South African HIV-infected patients. J Acquir Immune Defic Syndr. 2011;57: 284–9. 10.1097/QAI.0b013e318221863f 21602696

[24] Manuthu EM, Joshi MD, Lule GN, Karari E. Prevalence of dyslipidemia and dysglycaemia in HIV infected patients. East Afr Med J. 2008;85: 10–7. 18543521 10.4314/eamj.v85i1.9600

[25] Mercier S, Gueye NFN, Cournil A, Fontbonne AA, Copin N, Ndiaye I, et al Lipodystrophy and metabolic disorders in HIV-1-infected adults on 4- to 9-year antiretroviral therapy in Senegal: a case-control study. J Acquir Immune Defic Syndr. 2009;51: 224–30. 10.1097/QAI.0b013e31819c16f4 19339897

[26] Ngatchou W, Lemogoum D, Ndobo P, Tiogou E, Nga E, Kouanfack C, et al Increased burden and severity of metabolic syndrome and arterial stiffness in treatment-naïve HIV+ patients from Cameroon. Vasc Health Risk Manag. 2013;9: 509–16. 10.2147/VHRM.S42350 24043942 PMC3772749

[27] Thompson V, Medard B, Taseera K, Chakera AJ, Andia I, Emenyonu N, et al Regional anthropometry changes in antiretroviral-naïve persons initiating a Zidovudine-containing regimen in Mbarara, Uganda. AIDS Res Hum Retroviruses. 2011;27: 785–91. 10.1089/AID.2010.0272 21128866 PMC3159125

[28] Hattingh Z, Walsh C. The metabolic profiles of HIV-infected and non-infected women in Mangaung, South Africa. South African J Clin Nutr. 2009;22: 23–28.

[29] Mbanya JCN, Motala A a, Sobngwi E, Assah FK, Enoru ST. Diabetes in sub-Saharan Africa. Lancet. Elsevier Ltd; 2010;375: 2254–66. 10.1016/S0140-6736(10)60550-8 20609971

[30] Msyamboza KP, Mvula CJ, Kathyola D. Prevalence and correlates of diabetes mellitus in Malawi: population-based national NCD STEPS survey. BMC Endocr Disord. 2014;14: 41 10.1186/1472-6823-14-41 24884894 PMC4018960

[31] Peck R, Mghamba J, Vanobberghen F, Kavishe B, Rugarabamu V, Smeeth L, et al Preparedness of Tanzanian health facilities for outpatient primary care of hypertension and diabetes: a cross-sectional survey. Lancet Glob Heal. Elsevier Ltd; 2014; 1–8. 10.1016/S2214-109X(14)70033-6 PMC4013553 24818084

[32] Peck R, Fitzgerald DW, Liautaud B, Deschamps MM, Verdier RI, Beaulieu ME, et al The feasibility, demand, and effect of integrating primary care services with HIV voluntary counseling and testing: evaluation of a 15-year experience in Haiti, 1985–2000. J Acquir Immune Defic Syndr. 2003;33: 470–5. 12869835 10.1097/00126334-200308010-00007

[33] Calza L, Masetti G, Piergentili B, Trapani F, Cascavilla A, Manfredi R, et al Prevalence of diabetes mellitus, hyperinsulinaemia and metabolic syndrome among 755 adult patients with HIV-1 infection. Int J STD AIDS. 2011;22: 43–5. 10.1258/ijsa.2010.010256 21364066

[34] Carr A, Samaras K, Thorisdottir A, Kaufmann GR, Chisholm DJ, Cooper DA. Diagnosis, prediction, and natural course of HIV-1 protease-inhibitor-associated lipodystrophy, hyperlipidaemia, and diabetes mellitus: a cohort study. Lancet. 1999;353: 2093–9. 10382692 10.1016/S0140-6736(98)08468-2

[35] Erlandson KM, Kitch D, Tierney C, Sax PE, Daar ES, Melbourne KM, et al Impact of randomized antiretroviral therapy initiation on glucose metabolism. AIDS. 2014;28: 1451–61. 10.1097/QAD.0000000000000266 24637543 PMC4167596

[36] McComsey GA, Kitch D, Sax PE, Tierney C, Jahed NC, Melbourne K, et al Associations of inflammatory markers with AIDS and non-AIDS clinical events after initiation of antiretroviral therapy: AIDS clinical trials group A5224s, a substudy of ACTG A5202. J Acquir Immune Defic Syndr. 2014;65: 167–74. 10.1097/01.qai.0000437171.00504.41 24121755 PMC3943548

